# Minimally Invasive Mitral Valve Replacement in the Gray Zone: Bioprosthetic vs. Mechanical Valves in Patients Aged 50–69 Years

**DOI:** 10.3390/jcm14186666

**Published:** 2025-09-22

**Authors:** Alexander Weymann, Sadeq Ali-Hasan-Al-Saegh, Sho Takemoto, Nunzio Davide De Manna, Jan Beneke, Lukman Amanov, Fabio Ius, Ruemke Stefan, Bastian Schmack, Alina Zubarevich, Aburahma Khalil, Arjang Ruhparwar, Jawad Salman

**Affiliations:** 1 Department of Cardiothoracic, Transplantation and Vascular Surgery, Hannover Medical School, Carl-Neuberg-Straße 1, 30625 Hannover, Germany; 2 Center for Transplantation Sciences, Department of Surgery, Massachusetts General Hospital and Harvard Medical School, Boston, MA 02115, USA; 3 Department of Cardiovascular Surgery, Kyushu University Graduate School of Medical Sciences, Fukuoka 812-8582, Japan

**Keywords:** minimally invasive mitral valve surgery, mechanical valve, bioprosthetic, mitral valve disease

## Abstract

**Background**: Mitral valve replacement presents considerable challenges in the field of cardiothoracic surgery, particularly in patients aged 50 to 69, where the decision between bioprosthetic and mechanical valves is critical. Nevertheless, the optimal selection of prosthetic valves for candidates within this age-related gray zone remains inadequately defined, necessitating a thorough evaluation of long-term outcomes and associated risks. **Objective**: This study aims to assess mid-term outcomes of MIMVR in patients aged 50 to 69, comparing reoperation rates, prosthesis-related morbidity, and overall survival between bioprosthetic and mechanical valves. While many prior studies on valve choice in patients aged 50 to 69 years are derived from sternotomy cohorts, the novelty of our work lies in the exclusive focus on patients undergoing minimally invasive techniques. **Methods**: A retrospective analysis was conducted in accordance with the *Strengthening the Reporting of Observational Studies in Epidemiology* (STROBE) guidelines, including 172 patients aged 50–69 years who underwent minimally invasive mitral valve replacement via right minithoracotomy at a high-volume center in Germany between 2011 and 2023. Of the 172 patients, 95 underwent MIMVR using biological prostheses, while 77 received mechanical prostheses. Comprehensive data on demographics, surgical procedures, and postoperative complications, as well as long-term outcomes, were analyzed. **Results**: With a mean follow-up of 7.1 years, early outcomes revealed no significant differences in 30-day mortality (7.4% for bioprosthetic vs. 2.6% for mechanical; *p* = 0.06). There was no significant differences in all-cause mortality at 1 year (8.4% vs. 3.9%; *p* = 0.22), 3-year (9.5% vs. 7.8%; *p* = 0.69), and 5-year (13.7% vs. 10.4%; *p* = 0.19), or at the longest follow-up (13.7% vs. 10.4%; *p* = 0.51). Kaplan–Meier analysis showed no significant difference in long-term survival between the groups (*p* = 0.5427). Postoperative arrhythmia occurred significantly more frequently in the biologic group compared to the mechanical group (18.9% vs. 6.5%; *p* = 0.01). **Conclusions**: For patients aged 50–69 undergoing MIMVR using a bioprosthetic or mechanical valve, the mid-term survival and incidence of reoperation and re-hospitalization were comparable up to 7 years. This provides evidence supporting the safe application of the MICS approach with either valve type in this gray-zone age group.

## 1. Introduction

Mitral valve (MV) disease remains a cornerstone challenge in cardiothoracic surgery, often requiring replacement when repair is unfeasible. The advent of minimally invasive mitral valve replacement (MIMVR) has significantly redefined operative paradigms, offering reduced surgical trauma, faster recovery, and improved cosmetic outcomes compared to conventional median sternotomy [1,2,3]. Despite these advances, the optimal prosthesis selection within this evolving surgical landscape remains inadequately delineated, particularly in the gray zone of patients aged 50 to 69 years.

This age group occupies a complex crossroads in clinical decision-making, wherein prosthesis selection requires a judicious balance between long-term durability and the risks associated with anticoagulation [4,5]. Bioprosthetic valves eliminate the need for lifelong anticoagulation but carry a heightened risk of structural valve deterioration and potential reoperation [4,5]. In contrast, mechanical valves provide superior durability but are associated with lifelong anticoagulation, as well as the attendant risks of hemorrhage and thromboembolism [4,5]. Although existing studies have evaluated prosthesis performance in broader populations, the specific impact of valve selection within the minimally invasive approach remains insufficiently characterized [5,6]. The unique attributes of MIMVR, including smaller incisions, modified hemodynamic conditions, and distinct postoperative recovery profiles, may influence prosthesis-related outcomes, warranting focused analysis beyond traditional surgical approaches [5,6].

Current European and American guidelines advocate for mechanical valve implantation in patients younger than 60 years and bioprosthetic valves for those aged 70 and older [7,8]. However, these recommendations predominantly reflect data from conventional surgical techniques and fail to adequately address the implications of prosthesis type within the context of MIMVR. The unique perioperative conditions associated with minimally invasive surgery including altered hemodynamic dynamics, limited surgical exposure, and differentiated recovery trajectories may influence the effectiveness and complication rates of implanted prosthetic valves in ways not captured by traditional studies [6]. Furthermore, the underrepresentation of MIMVR-specific data for patients aged 50–69 further amplifies the uncertainty surrounding optimal prosthesis selection in this age group.

With a steadily aging population and the continuous evolution of surgical technologies, understanding the intersection between prosthesis choice and surgical technique has become paramount [6,7,8]. Long-term outcomes are especially relevant in the context of bioprosthetic valve degeneration and potential reoperation, while the bleeding risks associated with lifelong anticoagulation for mechanical valves must also be carefully weighed [6,7,8].

This retrospective single-center study therefore seeks to address this critical knowledge gap by evaluating mid-term outcomes such as reoperation rates, prosthesis-related morbidity, and survival outcomes following MIMVR in patients aged 50 to 69 years.

## 2. Materials and Methods

### 2.1. Study Population

From 2011 to 2023, our center in Germany performed a total of 934 mitral valve replacements through minimally invasive access. Among these, 172 procedures were conducted on patients aged 50 to 69, categorized as being in an age-related gray zone for surgical intervention. Of the 172 patients, 95 underwent MIMVR using biological prostheses, while 77 received mechanical prostheses.

Perioperative and postoperative variables were retrieved from the institutional database in a systematic manner. Eligible patients included those undergoing surgery for any etiology of mitral valve disease—degenerative, ischemic, rheumatic, or infective. Individuals were excluded if they had advanced extracardiac arteriopathy precluding safe femoral vessel cannulation for cardiopulmonary bypass (CPB) or if they presented with markedly impaired left ventricular ejection fraction.

### 2.2. Choice of Prosthesis

Prosthesis selection was primarily guided by a shared decision-making process between the surgeon and the patient. Patient preference played a key role, particularly regarding lifestyle considerations, while the operator’s judgment was also essential in cases with specific anatomical or clinical factors.

### 2.3. Surgical Approaches

The surgical techniques employed, including the right minithoracotomy approach, perfusion strategies, and aortic clamping techniques, have been previously detailed by our team [9,10]. All operations were performed via a right minithoracotomy, with continuous carbon dioxide insufflation applied during the entire procedure. Cardiopulmonary bypass was established through the cannulation of the right femoral vessels; the procedure was performed under single-lung ventilation [9].

Under echocardiographic guidance, venous drainage was first established by inserting a two-stage cannula into the superior vena cava, followed by arterial cannulation. The pericardium was then opened approximately 3–4 cm above the phrenic nerve. To achieve an activated clotting time exceeding 450 s, unfractionated heparin was administered intravenously, which was subsequently reversed with protamine after the removal of the venous cannula [9,10].

### 2.4. Data Collection and Follow-Up

Demographic characteristics, echocardiographic findings, operative details, and clinical outcomes were systematically recorded for all patients. Postoperative complications of interest included new-onset arrhythmias such as atrial fibrillation (NOAF), myocardial infarction, right ventricular failure, the requirement for permanent pacemaker implantation, thromboembolic events, acute renal failure requiring dialysis, respiratory insufficiency, major bleeding that necessitated re-thoracotomy, pneumothorax, ischemic stroke, delirium, intracranial hemorrhage, wound dehiscence, and sepsis.

Other metrics assessed included the length of intubation, the duration of catecholamine treatment, and the administration of blood cell concentrates or blood products. Early postoperative mortality was defined as any death occurring during the index hospitalization or within 30 days after surgery. We tracked all-cause mortality, freedom from mitral valve re-operation, and hospitalization due to major cardiovascular events, including heart failure, arrhythmias, and stroke, until February 2025.

### 2.5. Echocardiographic Assessment

The echocardiographic parameters recorded included left ventricular ejection fraction (LVEF); levels of mitral valve insufficiency (MI) classified as II, III, and IV; and degrees of mitral valve stenosis (MS) rated as II and III.

### 2.6. Ethical Statement

Following local German guidelines, the institutional ethical review board determined that approval was not necessary due to the study’s retrospective and non-interventional design.

### 2.7. Statistical Analysis

Analysis was conducted using SAS Enterprise Guide version 7.13. Continuous variables were summarized as means with standard deviations (SD) or as medians with interquartile ranges, depending on distribution. Categorical variables were reported as absolute counts and percentages. The Shapiro–Wilk and Kolmogorov–Smirnov tests were applied to determine whether continuous data followed a parametric or non-parametric distribution. Group comparisons were performed by Wilcoxon Rank-Sum Test for non-parametric variables and *t*-Test for parametric variables. Chi-square tests were used to assess categorical comparisons. Survival analysis for all-cause mortality was performed using Kaplan–Meier methods, and survival curves were plotted for the longest follow-up period with events. A *p*-value of less than 0.05 was considered statistically significant, and all statistical tests were conducted in a two-sided manner.

## 3. Results

### 3.1. Patient Characteristics

A total of 172 patients aged 50–69 undergoing MIMVR were included in the analysis. Of these, 95 (55.2%) received biologic prostheses and 77 (44.8%) received mechanical prostheses. The preoperative patient characteristics are outlined in Table 1. The mean age was comparable between the biologic and mechanical prosthesis groups (*p* = 0.72). There was no significant difference in the proportion of female patients. The prevalence of most baseline comorbidities was similar between the groups, including preoperative overall atrial fibrillation (AF) and its subtypes. A significantly higher proportion of patients receiving biologic prostheses had a history of coronary artery disease compared to those receiving mechanical prostheses. The rates of previous cardiac surgery (re-operation) were not statistically different between the groups. The preoperative NYHA functional class was comparable and the preoperative use of amiodarone, betablockers, calcium channel blockers, and digoxin also showed no statistically significant differences between the groups.

### 3.2. Procedural Characteristics

Intraoperative data are presented in Table 2. The median duration of surgery was significantly longer for patients receiving biologic prostheses compared to mechanical prostheses. However, the median time on cardiopulmonary bypass (CPB) was similar between the two groups. Concomitant tricuspid valve repair and Maze procedure were performed at comparable rates in both groups. Left atrial appendage occlusion (LAAO) was performed significantly more often in the biologic prosthesis group than in the mechanical prosthesis group. Chordae tendineae rupture was observed in similar proportions in both groups.

### 3.3. Echocardiographic Assessments

The preoperative and postoperative echocardiographic findings are described in Table 3. Preoperatively, the mean left ventricular ejection fraction (LVEF) was similar between the biologic and mechanical prosthesis groups (48.4 ± 19.1% vs. 49.2 ± 18.7%; *p* = 0.99). The distribution of preoperative mitral insufficiency (MI) severity did not differ significantly between groups, with Grade III MI being the most common finding in both cohorts (81.3% vs. 70.3%; *p* = 0.12). Regarding preoperative mitral stenosis (MS), patients in the mechanical prosthesis group had a significantly higher prevalence of Grade III MS compared to the biologic group (2.5% vs. 13.8%; *p* = 0.01). No patients had myxoma or fibroma.

Postoperatively, mean LVEF remained comparable between the biologic and mechanical groups (49.6 ± 11.7% vs. 50.4 ± 14.2%; *p* = 0.74). No patients in either group had postoperative MI Grade III or IV, or MS Grade III. Residual MI Grade I (11.6% vs. 13.0%; *p* = 0.77) and MI Grade II (1.1% vs. 0%; *p* = 0.36) were infrequent in both groups. Only one patient (1.3%) in the mechanical group exhibited postoperative MS Grade II, with no significant difference between the groups (*p* = 0.26).

### 3.4. Early Outcomes

The early postoperative outcomes are summarized in Table 4. There were no significant differences in the in-hospital mortality and 30-day mortality (6 [6.3%] and 7 [7.4%] vs. 2 [2.6%] and 2 [2.6%]; *p* = 0.24, 0.06, respectively). The mean duration of catecholamine therapy (78.2 ± 140.5 vs. 51.68 ± 110.21 h; *p* = 0.43) and the mean units of blood transfusion were comparable between the groups. Postoperative arrhythmia occurred significantly more frequently in the biologic group compared to the mechanical group (18.9% vs. 6.5%; *p* = 0.01). The rates of other early complications were similar between the groups, including early mitral valve re-operation (2.1% vs. 1.3%; *p* = 0.68), major bleeding (12.6% vs. 11.7%; *p* = 0.85), new-onset AF (11.6% vs. 10.4%; *p* = 0.80), stroke (3.2% vs. 2.6%; *p* = 0.82), thromboembolic events (1.1% vs. 3.9%; *p* = 0.21), and myocardial infarction (1.1% vs. 2.6%; *p* = 0.44). No considerable prosthesis–patient mismatch was observed in either the bioprosthetic or mechanical valve groups.

### 3.5. Late Outcomes

**Figure 1:**
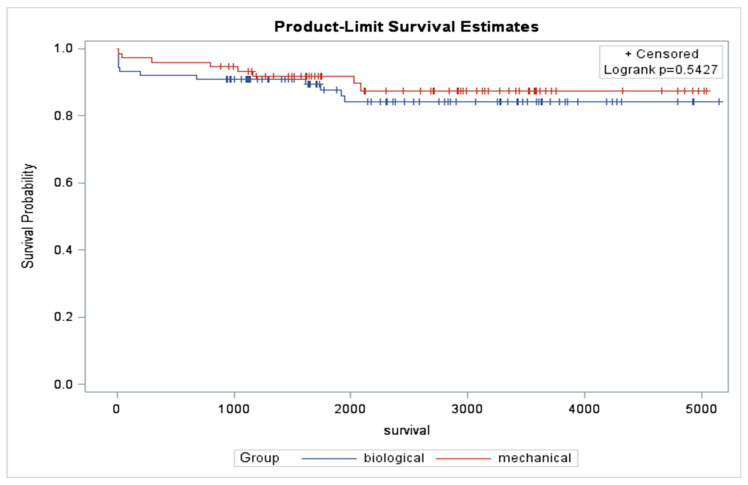
Long-term survival.

The mean follow-up durations were 7.1 ± 3.1 years for the biologic group and 7.3 ± 3.1 years for the mechanical group (*p* = 0.79). Long-term follow-up revealed no significant differences in all-cause mortality—at 1 year, mortality rates were 8.4% in the biologic group compared to 3.9% in the mechanical group (*p* = 0.22); at 3 years, 9.5% vs. 7.8% (*p* = 0.69); at 5 years, 13.7% vs. 10.4% (*p* = 0.19); and at the longest follow-up, 13.7% vs. 10.4% (*p* = 0.51) (Table 5). Kaplan–Meier analysis also indicated no significant difference in long-term survival between the groups (*p* = 0.5427; Figure 1).

In terms of late mitral valve re-operation, seven patients (7.4%) in the biologic group and seven patients (9.1%) in the mechanical group underwent this procedure during the follow-up period (*p* = 0.68). Furthermore, long-term re-hospitalization rates for specific cardiovascular causes were comparable between the groups. Re-hospitalization due to arrhythmia occurred in 17.9% vs. 19.5% (*p* = 0.79), heart failure in 20.0% vs. 22.1% (*p* = 0.73), myocardial infarction in 1.1% vs. 2.6% (*p* = 0.44), and stroke in 4.3% vs. 5.2% (*p* = 0.77) (Table 5).

## 4. Discussion

While many prior studies on valve choice in patients aged 50 to 69 years are derived from sternotomy cohorts, the novelty of our work lies in the exclusive focus on patients undergoing minimally invasive techniques. This single-center, retrospective study investigated patients aged 50 to 69 years undergoing MIMVR using either biologic or mechanical prostheses and highlights comparable early and late outcomes. Mitral valve repair is generally regarded as the preferred treatment, especially in cases of degenerative disease. In our study population, however, all patients proceeded directly to valve replacement without an initial repair attempt. This approach was determined by the specific clinical profiles and intraoperative findings, which indicated that repair would not have provided a durable or reliable result. In the early postoperative period, no significant difference in 30-day mortality was noted (biologic: 7.4% vs. mechanical: 2.6%; *p* = 0.06). Major complications, including new-onset AF, bleeding, and stroke were also similar. Crucially, in the late outcomes, with a mean follow-up extending beyond 7 years, there were no significant differences between the two groups in all-cause mortality, freedom from mitral valve re-operation, or re-hospitalization for major cardiovascular causes including heart failure, arrhythmia, and stroke. The EUROScore was not calculated for our cohort; therefore, a risk-adjusted analysis could not be performed. We recognize this as a limitation and suggest that future studies should incorporate EUROScore assessment to better account for baseline surgical risk.

Our findings on survival align partially with previous studies, while the results regarding reoperation differ. Fialka et al., utilizing the Alberta provincial database for patients aged 50–70 undergoing MVR via median sternotomy, reported similar survival between biologic and mechanical valve recipients but a significantly higher rate of mitral valve re-replacement in the biologic group at a median follow-up of 10.7 years [11]. Similarly, Goldstone et al. and Chikwe et al. also reported comparable survival between the two groups aged 50 to 69 years [4,5]. On the other hand, Kaneko et al. reported that biologic MVR for patients < 65 years old was associated with a high reoperation rate and decreased survival [12]. Building on these studies, our study focused specifically on MIMVS within a single institutional cohort. With mean follow-up durations of approximately 7.1 years for both groups, we observed no significant differences in all-cause mortality across various time points, including 1-year, 3-year, and 5-year intervals. These findings suggest that both types of valves offer comparable survival benefits in the long term. The Kaplan–Meier analysis further corroborated these results, indicating no significant difference in long-term survival between the groups. This is particularly noteworthy given the low mortality rates observed, which may reflect advancements in surgical techniques and postoperative care over recent years. Additionally, our findings related to late mitral valve re-operation were consistent across both groups, with similar rates observed. The re-hospitalization rates for specific cardiovascular events were also comparable, reinforcing the notion that both valve types perform similarly in terms of long-term complications. We observed no significant difference in survival between the groups, which is consistent with the survival findings of Fialka’s study, although our follow-up duration is shorter. In contrast, a meta-analysis by Yanagawa et al. involving MVR patients younger than 70 years reported significantly lower postoperative mortality, long-term mortality (median 8 years), and MVR reoperation rates after mechanical MVR [13]. Similarly, another meta-analysis by Ahmed et al. indicated that mechanical mitral valve use was associated with reduced 30-day and long-term mortality over a mean follow-up of 14 years [6]. Regarding reoperation, Fialka’s [11], Chikwe’s [5], and our study featured a lower age limit of 50 years; however, in contrast, we did not find a significant difference in the incidence of reoperation between the groups. This discrepancy may reflect the shorter follow-up duration in our study (7.1 years), suggesting the need for continued observation to assess late valve deterioration. While previous studies were limited by the inclusion of patients younger than 50 [4,12], those who may experience earlier structural valve deterioration (SVD), or did not differentiate between median sternotomy and MICS approaches, our analysis, despite its smaller cohort size, provides important insights specific to the contemporary practice where MICS is frequently used for MVR in this age group.

Another notable finding, differing from previous reports that have associated mechanical valves with lower new-onset AF (NOAF) risk [11], is the lack of a significant difference in the incidence of NOAF between the groups. NOAF is recognized as a significant complication associated with increased risks of mid- to long-term stroke and mortality after mitral valve surgery [14,15]. It has been suggested that median sternotomy itself can be a risk factor for NOAF [16]. Therefore, the exclusive use of the MICS approach for all patients in both our groups may have contributed to mitigating AF development overall. Supporting the less invasive nature of the procedures in our cohort, the median operative times (biologic: 227 min [IQR 191–263]; mechanical: 209 min [IQR 174–242]) compare favorably to the median 3–6 h reported in the sternotomy cohort by Fialka et al. [11]. Consistent with the similar postoperative NOAF incidence, we observed no significant differences in either early postoperative stroke or late stroke-related re-hospitalization between the biologic and mechanical valve recipients. While preoperative LVEF and the use of beta-blockers were comparable between the groups, it is important to note that the extent of preoperative left atrial enlargement was not specifically compared, and left atrial appendage occlusion was performed significantly more often concomitantly in the biologic valve group, which could potentially influence thromboembolic outcomes.

Current guidelines from the American Heart Association/American College of Cardiology (AHA/ACC) and the European Society of Cardiology/European Association for Cardio-Thoracic Surgery (ESC/EACTS) recommend mechanical prostheses for patients younger than 60 years and biologic prostheses for those older than 70 years [7,8]. However, both guidelines acknowledge a “gray zone” for patients within this intermediate age range, emphasizing the need for individualized decision-making based on patient-specific factors. This requires a shared decision-making process that carefully balances the anticipated lifetime risk of SVD and potential reoperation with a biologic valve against the risks of bleeding and thromboembolic events associated with lifelong anticoagulation for a mechanical valve. Other critical considerations include the patient’s estimated life expectancy, medication adherence, and their personal preferences and lifestyle. Furthermore, while the potential need for reoperation with biologic valves has historically been a concern regarding long-term survival [4,12], efforts are ongoing to improve the durability of biologic prosthesis [17]. Additionally, the advent of transcatheter mitral valve-in-valve replacement presents evolving options for managing SVD, potentially altering the paradigm of lifetime valve management [18]. Consequently, determining the optimal valve prosthesis for patients within this gray zone age group is becoming increasingly complex. The present study, which focused on the MIMVR population, may provide valuable evidence suggesting that both biologic and mechanical valves represent safe and viable options with similar mid-term results in patients aged 50–70, thereby supporting the feasibility and safety of either choice when utilizing this increasingly common minimally invasive approach. Valve-in-valve procedures using transcatheter techniques may serve as a reliable strategy for managing bioprosthetic valve failure after MIMVR.

This study has several important limitations. It represents a single-center, retrospective experience, and the absence of randomization introduces the possibility of selection bias. Decisions regarding valve type were primarily based on patient characteristics and surgeon preference, which may have influenced outcomes. Furthermore, formal risk adjustment using established scoring systems such as the EUROScore was not performed, limiting comparability with other published series. We suggest that future studies should evaluate the surgical risk of patients using the EUROScore. All patients in this cohort underwent valve replacement rather than attempted repair, as repair was not considered feasible in these cases. Another limitation is the relatively limited follow-up regarding bioprosthetic durability. Although the mean follow-up of around seven years offers meaningful mid-term insight, it may not be sufficient to fully evaluate late complications such as structural valve deterioration, bleeding or thromboembolic events, and prosthetic valve endocarditis.

## 5. Conclusions

In patients aged 50–69 undergoing MIMVR using bioprosthetic or mechanical valves, the mid-term survival and incidence of reoperation and re-hospitalization were comparable up to 7 years. MIMVR procedures with either prosthesis are safe in the mid-term; however, prosthesis selection remains complex and should be individualized. Recognizing the primacy of mitral valve repair and the necessity of longer follow-up will provide a more balanced and clinically relevant perspective.

## Figures and Tables

**Table 1:** Preoperative data.

Variables	Biologic Prostheses N = 95	Mechanical Prostheses N = 77	*p*-Value
Age	65.4 (8.1)	61.0 (6.8)	0.72
Female	37 (38.9%)	38 (49.4%)	0.17
History of renal disease	25 (26.3%)	16 (20.8%)	0.39
History of dialysis	9 (9.5%)	6 (7.8%)	0.65
Hyperlipidemia	49 (51.6%)	32 (41.6%)	0.19
Hypertension	67 (70.5%)	55 (71.4%)	0.89
Insulin-dependent diabetes mellitus	7 (7.4%)	7 (9.1%)	0.68
History of smoking	28 (29.5%)	27 (35.1%)	0.43
Obesity	28 (29.5%)	27 (35.1%)	0.40
Atrial fibrillation	50 (52.6%)	45 (58.4%)	0.44
Paroxysmal	18 (18.9%)	15 (19.5%)	0.92
Permanent	18 (18.9%)	13 (16.9%)	0.72
Persistent	14 (14.7%)	17 (22.1%)	0.21
Re-operation	23 (24.2%)	26 (33.8%)	0.16
Active endocarditis	10 (10.5%)	8 (10.4%)	0.97
History of coronary artery disease	41 (43.2%)	20 (26%)	0.01
History of periphery artery disease	13 (13.7%)	10 (13%)	0.89
Stroke	17 (17.9%)	10 (13%)	0.37
Pulmonal hypertension	54 (56.8%)	43 (55.8%)	0.89
Recent myocardial infarction	2 (2.1%)	2 (2.6%)	0.83
Elective operation	56 (58.9%)	49 (63.6%)	0.53
Urgent operation	32 (33.7%)	22 (28.6%)	0.47
Emergency operation	7 (7.4%)	6 (7.8%)	0.91
**NYHA**			
Class I	1 (1.1%)	2 (2.6%)	0.44
Class II	31 (32.6%)	24 (31.2%)	0.83
Class III	48 (50.5%)	40 (51.9%)	0.85
Class IV	7 (7.4%)	4 (5.2%)	0.56
Preoperative drug			
Amiodarone	2 (2.1%)	4 (5.2%)	0.27
Beta blocker	65 (68.4%)	58 (75.3%)	0.31
Calcium channel blocker	11 (11.6%)	16 (20.8%)	0.09
Digoxin	9 (9.5%)	14 (18.2%)	0.09

Values are *n* (%), median (25%tile–75%tile), or mean (SD).

**Table 2:** Procedure characteristics.

Variables	Biologic Prostheses N = 95	Mechanical Prostheses N = 77	*p*-Value
Duration of surgery (minutes)	227 (191–263)	209 (174–242)	0.03
Time on CPB (minutes)	147 (113–188)	139 (118–173)	0.33
Left atrial appendage occlusion	28 (29.5%)	12 (15.6%)	0.03
Combined tricuspid valve repair	17 (17.9%)	8 (10.4%)	0.16
Maze procedure	21 (22.1%)	11 (14.3%)	0.19
Chordae tendineae rupture	13 (13.7%)	11 (14.3%)	0.90

Values are *n* (%), median (25%tile–75%tile), or mean (SD). CPB: cardiopulmonary bypass.

**Table 3:** Echocardiographic assessments.

Time	Variables	Biologic Prostheses N = 95	Mechanical Prostheses N = 77	*p*-Value
Preoperative	LVEF (%)	48.4 (19.1)	49.2 (18.7)	0.99
	MI II	10 (13.3%)	15 (23.8%)	0.11
	MI III	61 (81.3%)	45 (70.3%)	0.12
	MI IV	4 (5.3%)	9 (14.1%)	0.07
	MS II	2 (2.5%)	5 (7.6%)	0.14
	MS III	2 (2.5%)	9 (13.8%)	0.01
	Myxoma	0 (0%)	0 (0%)	1
	Fibroma	0 (0%)	0 (0%)	1
Postoperative	LVEF (%)	49.6 (11.7)	50.4 (14.2)	0.74
	MI I	11 (11.6%)	10 (13%)	0.77
	MI II	1 (1.1%)	0 (0%)	0.36
	MI III	0 (0%)	0 (0%)	1
	MI IV	0 (0%)	0 (0%)	1
	MS II	0 (0%)	1 (1.3%)	0.26
	MS III	0 (0%)	0 (0%)	1

Values are *n* (%), median (25%tile–75%tile), or mean (SD). LVEF: left ventricular ejection fraction; MI: mitral insufficiency; MS: mitral stenosis.

**Table 4:** Early outcomes.

Variables	Biologic Prostheses N = 95	Mechanical Prostheses N = 77	*p*-Value
In-hospital mortality	6 (6.3%)	2 (2.6%)	0.24
30-day mortality	7 (7.4%)	2 (2.6%)	0.06
Duration of therapy with catecholamine (minutes)	78.2 (140.5)	51.7 (110.2)	0.43
Blood transfusion (units)			
FFP	2 (0–4)	0 (0–3)	0.57
Erythrocyte	3 (2–7)	3 (2–6)	0.77
Platelet	0 (0–1)	0 (0–0)	0.17
Respiratory insufficiency	17 (17.9%)	7 (9.1%)	0.09
Early mitral valve re-operation	2 (2.1%)	1 (1.3%)	0.68
Arrhythmia	18 (18.9%)	5 (6.5%)	0.01
ECMO/right ventricular failure	11 (11.6%)	6 (7.8%)	0.40
Re-thoracotomy	12 (12.6%)	9 (11.7%)	0.85
Major bleeding	12 (12.6%)	9 (11.7%)	0.85
New-onset atrial fibrillation	11 (11.6%)	8 (10.4%)	0.80
Renal failure with new-onset dialysis	11 (11.6%)	4 (5.2%)	0.14
Stroke	3 (3.2%)	2 (2.6%)	0.82
Cerebral bleeding	0 (0%)	0 (0%)	1
Seizure	3 (3.2%)	2 (2.6%)	0.82
Delirium	3 (3.2%)	2 (2.6%)	0.82
Thromboembolic events	1 (1.1%)	3 (3.9%)	0.21
Wound dehiscence	7 (7.4%)	9 (11.7%)	0.33
Sepsis	4 (4.2%)	1 (1.3%)	0.25
Myocardial infarction	1 (1.1%)	2 (2.6%)	0.44
Pacemaker implantation	5 (5.3%)	2 (2.6%)	0.37
Pneumothorax	7 (7.4%)	2 (2.6%)	0.16

Values are *n* (%), median (25%tile–75%tile), or mean (SD). ECMO: extracorporeal membrane oxygenation; FFP: fresh frozen plasma.

**Table 5:** Late outcomes.

Variables	Biologic Prostheses N = 95	Mechanical Prostheses N = 77	*p*-Value
Follow-up (years)	7.1 (3.1)	7.3 (3.1)	0.79
Late mortality			
1 year mortality	8 (8.4%)	3 (3.9%)	0.22
3-year mortality	9 (9.5%)	6 (7.8%)	0.69
5-year mortality	13 (13.7%)	8 (10.4%)	0.19
At longest follow-up	13 (13.7%)	8 (10.4%)	0.51
Late mitral valve re-operation	7 (7.4%)	7 (9.1%)	0.68
Re-hospitalization			
Arrhythmia	17 (17.9%)	15 (19.5%)	0.79
Heart failure	19 (20%)	17 (22.1%)	0.73
Myocardial infarction	1 (1.1%)	2 (2.6%)	0.44
Stroke	4 (4.3%)	4 (5.2%)	0.77

Values are *n* (%) or mean (SD).

## Data Availability

Data are contained within the article.

## Abbreviations

The following abbreviations are used in this manuscript:

MIMVR	Minimally invasive mitral valve replacement
MV	Mitral valve
MI	Mitral valve insufficiency
MS	Mitral valve stenosis
CPB	Cardiopulmonary bypass
NOAF	New-onset arrhythmias and atrial fibrillation
LVEF	Left ventricular ejection fraction
SD	Standard deviation
AF	Atrial fibrillation
NYHA	New York Heart Association
ECMO	Extracorporeal membrane oxygenation
FFP	Fresh frozen plasma
SVD	Structural valve deterioration
AHA/ACC	American Heart Association/American College of Cardiology
ESC/EACTS	European Society of Cardiology/European Association for Cardio-Thoracic Surgery
MICS	Minimally invasive cardiac surgery
